# The Pathogenesis of Lupus Nephritis

**DOI:** 10.4172/2155-9899.1000205

**Published:** 2014-04

**Authors:** Rosalie M. Sterner, Stella P. Hartono, Joseph P. Grande

**Affiliations:** 1 Department of Laboratory Medicine & Pathology, Mayo Clinic, 200 First Street SW, Rochester, MN 55905 USA; 2 Medical Scientist Training Program, Mayo Clinic, 200 First Street SW, Rochester, MN 55905 USA

**Keywords:** Kidney, Lupus, Nephritis, Immune complex, Mechanism

## Abstract

Lupus nephritis is a serious potential feature of systemic lupus erythematous (SLE). Though SLE typically cycles through periods of flares and remission, patients often eventually succumb to end-stage kidney or cardiovascular damage. This review of the pathogenesis of lupus nephritis examines the role of the complement cascade; the significance of autoantibodies, the breaking of tolerance, and the implications of altered apoptosis in breaking tolerance; and the contributions of adaptive immunity and cross-talk with the innate immune system in driving renal damage. Delineation of basic mechanisms underlying the development of acute and chronic renal damage in lupus nephritis can result in the continued development of more specific and effective treatments.

## Introduction

**Figure 1:**
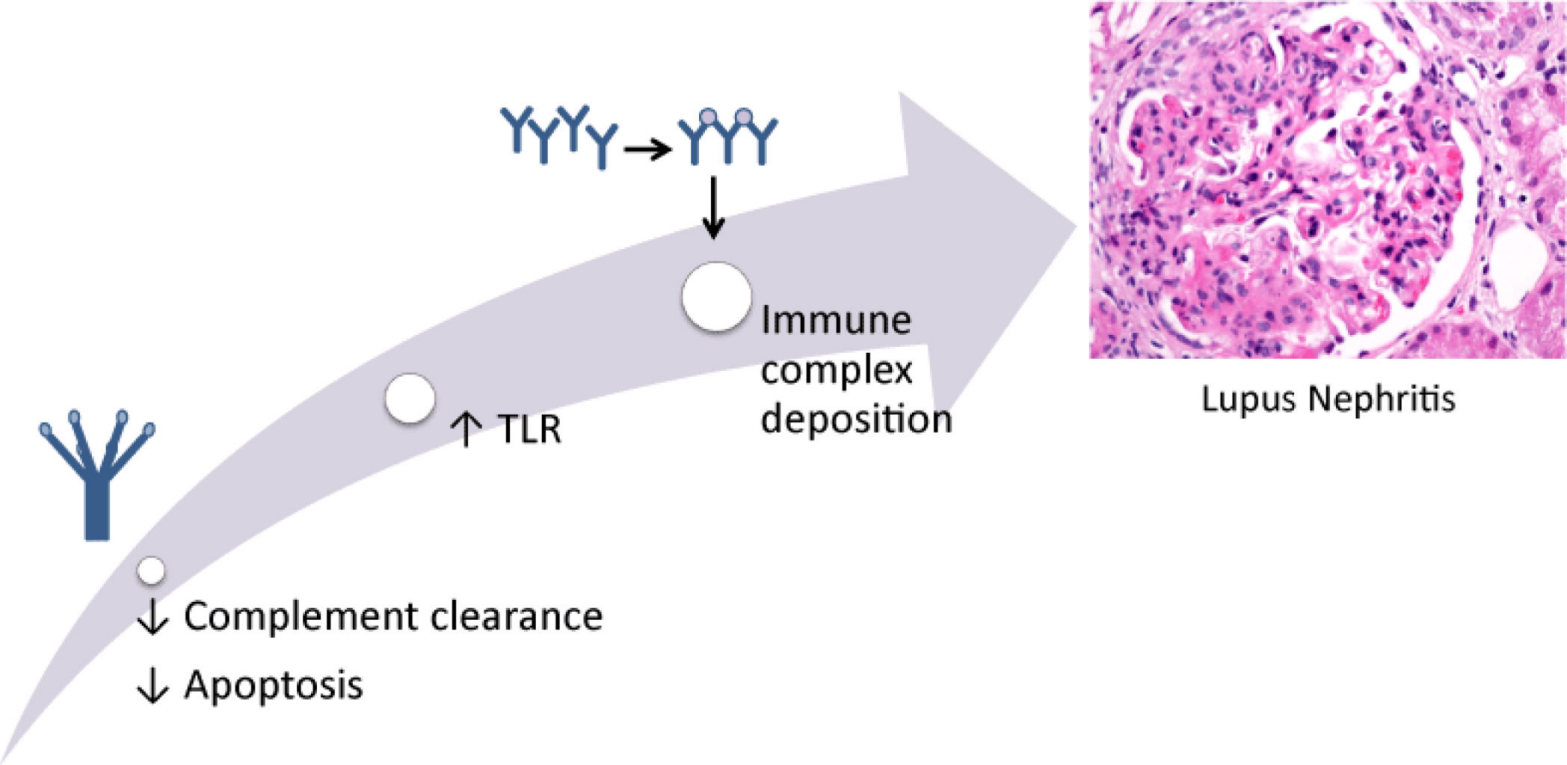
Proposed mechanism of immune complex deposition of lupus nephritis.

Lupus nephritis is a serious potential feature of systemic lupus erythematous (SLE). Systemic lupus erythematous can affect numerous organs ranging from the kidneys, skin, pericardium, lungs, nervous system and beyond [1]. Though SLE typically cycles through periods of flares and remission, patients often eventually succumb to end-stage kidney or cardiovascular damage [1]. The incidence of SLE in women is nine times higher than in men [2]. Renal manifestations may be the first symptomatic finding often within one year of diagnosis but usually sometime within the first five years after diagnosis [2]. SLE is characterized by the presence of numerous autoantibodies that can form immune complexes whose deposition in the kidneys contributes greatly to the pathogenesis of lupus nephritis [2]. This review of the pathogenesis of lupus nephritis examines the role of the complement cascade; the significance of autoantibodies, the breaking of tolerance, and the potential implications of altered apoptosis in breaking tolerance; and the contributions of adaptive immunity and cross-talk with the innate immune system in driving renal damage (Figure 1).

## Complement and Lupus Nephritis

Complement is a component of the innate immune response that aids in opsonizing immune complexes for degradation by effector immune cells [3]. As lupus nephritis is characterized by renal deposition of these immune complexes, complement is believed to play a significant role in its pathogenesis [3–5]. There are three complement pathways: the classical pathway, the alternative pathway, and the lectin pathway [3,4]. With concern to lupus nephritis and SLE, the classical pathway is believed to be the most significant and has been most widely studied in the context of SLE [3,4]. In the classical pathway, the C1 complex binds to the Fc region of the IgG antibodies of immune complexes [3,4]. The C1 complex contains C1q, which consists of three polypeptides each with six collagen like stalks that link together the fibril central region with the six globular heads of C1q [6,7]. The C1q collagen like stalks are where C1r and C1s can dock in order to produce a C1 complex capable of binding the IgG Fc region [3,4]. The bound C1 complex is then capable of cleaving C4 and C2 in order to form a C3 convertase complex that can cleave C3 into C3a and C3b. C3b aids in opsonizing material for phagocytosis and the clearance of the immune complexes [3,4]. In the presence of C3b, C3 convertases can form C5 convertases, which cleave C5 to C5a and C5b. C5a is a powerful chemotactic agent that can aid in the recruitment of inflammatory cells such as neutrophils, eosinophils, monocytes, and T lymphocytes, and it can stimulate phagocytosis of cells and release of reactive oxygen species that can damage host tissue [8]. C5b is necessary for the formation of the C5b-9 membrane attack complex, which can penetrate cell membranes and lyse cells [3].

Genetic deficiency of C1, and to some extent C2 and C4, can result in a strong propensity to develop severe SLE mostly likely due to the diminished capacity of the immune system to clear immune complexes [5,9]. Hereditary C1q deficiency is very rare and can either occur as a nonsense mutation where no protein is present or a missense mutation where C1q is nonfunctional [5]. The penetrance of these gene mutations is very high and seen across a wide array of genetic backgrounds, which indicates that early complement may play a key role in guarding against SLE, though it is quite interesting that deficiencies at the convergence point of the three complement pathways, C3, rarely manifests in SLE [5].

While heritable mutations of C1q leading to deficiency are rare, deficiency due to excessive complement activation because of interaction with immune complexes is common, and low C1q levels are associated with active disease [5]. In SLE, the size of circulating immune complexes has been tied to glomerulonephritis and interstitial nephritis [10]. Confounding this is the observation that patients often develop autoantibodies to C1q [5]. When C1q binds an immune complex, its change in conformation results in the exposure of new antigenic sites [11,12]. Autoantibodies are then free to bind the newly exposed collagen-like region of C1q [13,14]. In MRL/lpr/lpr mice, a model for SLE, an age-dependent downward trend of serum C1q levels were correlated with an increase of autoantibody against C1q, implying a link with glomerulonephritis through consumption of C1q through complex formation [15]. There is a positive correlation between the presence of antibody against C1q and clinical findings including nephritis, dermatitis, hypocomplementemia, dsDNA antibodies, and circulating immune complexes. The concentration of autoantibodies to C1q in the glomeruli has been observed to be greater than or equal to fifty fold above those found in the serum, this deposition likely contributing to the pathogenesis of lupus nephritis [16]. Six months prior to the appearance of clinical renal signs, serum titres of IgG autoantibodies are typically increased [17,18]. This indicates their importance as a potential tool for predicting a renal episode of proliferative lupus nephritis [18]. In the peripheral blood, there is an increase in anti-C1q antibodies in active renal disease, which then returns to normal after treatment-induced remission [19–24]. The presence of both hematuria and anti-C1q antibodies potentially correlate to current inflammation in the glomeruli in lupus [25]. With respect to one category of antibodies, anti-C1q autoantibodies are believed to be most strongly associated with renal disease activity, and both anti-C1q and anti-dsDNA in combination are tied to increased renal disease activity and poor renal outcomes [26,27].

There is evidence that there can also be anti-C1q autoantibodies capable of binding the globular head of C1q [28]. The autoantibodies against the globular head region of the B chain of C1q inhibited C1q interaction with IgG and C reactive protein, which could result in a decrease in functional C1q [28].

While the classical pathway is the foundation of pathogenesis of lupus nephritis, the alternative pathway and lectin pathway appear to play a role in the progression of glomerular damage [29]. Patients presenting with the glomerular deposition of properdin, a positive regulator of the alternate pathway, and patients with Mannose binding lectin/L-ficolin showed increased urinary protein excretion [29].

However, immune complex formation and complement activation alone are not enough to generate lupus nephritis [30]. Disruption of the gamma chain of the Fc receptor in New Zealand Black/New Zealand White prevented severe glomerulonephritis while immune complex deposition still occurred [30]. In humans, the heritable susceptibility factor to SLE of Fc gamma receptor type IIA R131 may cause failure of clearance of an IgG2 anti-C1q antibody to the collagen like region of C1q complex leading to glomerulonephritis [31]. In Egyptian SLE patients, there is an association of an Fc gamma type IIB homozygous genotype (Thr/Thr) with increased risk of SLE and complications of nephritis, but the Fc receptor type IIA polymorphisms were not associated in Egyptian patients [32]. Recombinant human soluble Fc gamma receptors (CD32) bound IgG coated murine erythrocytes, decreasing phagocytosis by macrophages via disruption of the IgG and Fc gamma receptor interaction [33]. While CD32 treatment did not reverse nephritis, there was a delayed onset of proteinuria and increased survival time [33]. A synthetic peptide from the membrane-proximal extracellular domain of Fc gamma receptor II was found to be a competitive inhibitor of IgG binding recombinant Fc receptor II *in vitro* and increased survival while decreasing proteinuria in MRL/lpr mice *in vivo* [34]. Taken together, glomerular deposition of C1q in the context of immune complexes, complement activation, and functional Fc gamma receptors appear to be necessary to cause renal damage [35].

## Autoantibodies and Apoptosis in Lupus Nephritis

Lupus nephritis is characterized by renal deposition of immune complexes. IgG antinuclear autoantibodies against components such as DNA and nucleoprotein are commonly found in the glomeruli and serum of individuals with lupus nephritis [36]. Circulating immune complex antibodies have been shown to more readily bind DNA but not glomerular basement membrane antigens whereas IgG from the glomeruli of SLE patients readily bound DNA, glomerular basement membrane antigen, proteoglycan, and heparan sulfate [37]. However, after treatment with heparitinase glomerular deposition of IgG was decreased, indicating potential direct glomerular basement membrane binding and immune complex formation through heparan sulfate by some anti-DNA autoantibodies [37]. *In vitro*, nucleosome and C1q deposition to glomerular endothelial cells is at least partially mediated by surface heparan sulfate and allows for subsequent binding of autoantibodies against nucleosomes that may be pathogenic and the autoantibodies against the C1q may further drive pathogenesis [38]. Conversely, after passage through Sepharose with glomerular basement membrane antigen, renal eluates lost the ability to bind glomerular basement membrane but still possessed the ability to bind DNA indicating a role for circulating immune complex glomerular deposition, suggesting that both mechanisms of deposition may play a role in lupus nephritis pathogenesis [37].

The ability to form immune complex depositions and where said immune complexes are formed varies based on the individual autoantibody involved [39]. In mouse models using various anti-DNA antibodies, mesangial and subendothelial immune complex depositions were correlated with proliferative glomerulonephritis, neutrophil infiltration, and proteinuria; diffuse fine granular mesangial and extraglomerular vascular immune complex depositions were correlated with proliferative glomerulonephritis and proteinuria; dense intramembranous and intraluminal immune complex depositions were correlated with thickening of the capillary walls, mesangial interposition, mesangial expansion, aneurysmal dilatation, blockage of the capillary loops of the glomeruli within the lumen, and extensive proteinuria; and mesangial and extraglomerular vascular immune complex deposition correlated with slight segmental mesangial expansion and no associated proteinuria [39].

It is suggested that initial breakdown of immune tolerance with chromatin may begin with autoantibodies targeting (H2A-H2B)-DNA complexes and that (H3-H4)_2_-DNA and double stranded DNA alone become targets only after further loss of tolerance [40]. In apoptosis of cells, small blebs at the surface of said cells have been found to contain pieces of the endoplasmic reticulum, ribosomes, and the ribonucleoprotein Ro and larger apoptotic bodies containing nucleosomal DNA, the ribonucleoprotein Ro, the ribonucleoprotein La, and small nuclear ribonucleoproteins [41]. These blebs are near the ER and nuclear membrane, which produce more reactive oxygen species and make oxidation of the blebs’ contents plausible, potentially encouraging a variety of different molecules to act as autoantigens due to the similar processing via oxidation [41]. In the context of viral infection, apoptotic cells can produce blebs with high concentrations of viral antigen and autoantigen, which may also challenge self-tolerance [42].

On average, a study by Arcbuckle et al. showed that autoantibodies are present 3.3 years before the presence of symptomatic SLE, and a general pattern of development was observed with antinuclear, antiphospholipid, anti-Ro, and anti-La presenting before anti-Sm and anti-ribonucleoprotein antibodies [43]. Anti-nuclear antibodies developed before anti-double-stranded DNA antibodies, which develops before anti-ribonucleoprotein antibodies [43]. Lupus nephritis has been associated with intraglomerular cell apoptosis in which nucleosomes are released in apoptosis and then associate with glomerulus basement membranes, which potentially allows these apoptotic nucleosomes to be both the inducer and target for the autoantibodies causing lupus nephritis in SLE [44]. Immune electron microscopy further demonstrated that anti-double-stranded DNA autoantibodies target apoptotic intra-glomerular membrane-associated nucleosomes [45].

Transforming growth factor-β1, which is expressed by podocytes and mesangial cells in lupus nephritis, can aid in the replacement of expression of laminin-11 normally found in the mature glomerular basement membrane with laminin-1, which is normally only expressed in development [46,47]. Nucleosomes readily bind to laminin-1 through the β1 chain but do not bind laminin-11, which lacks the β1 chain, and the trapped nucleosomes can then be bound by autoantibodies that rev up t-cell-dependent autoimmune responses, adding insight into the early pathogenesis of lupus nephritis [46,47]. Cross reactivity of monoclonal antibodies that can bind both double-stranded DNA and glomerular antigen α-actinin has high affinity; however, in kidney sections with lupus nephritis autoantibodies did not readily bind those components but preferentially bound to nucleosome-containing structures in the mesangial matrix or glomerular basement membrane, and nucleosomes most readily bound to laminin [48].

In SLE, noninflammatory phagocytosis of apoptotic cells is decreased, allowing apoptotic contents to circulate and potentially initiate inflammatory removal pathways, stimulate self-reactive lymphocytes, and encourage immune complexes to form [49]. In some SLE patients, apoptotic cells have been observed to build up in germinal centers where the number of tangible body macrophages, which are capable of processing the nuclear contents of apoptotic cells, were decreased, and this could allow self-antigens from the apoptotic nuclei to interact with follicular dendritic cells, inducing survival signals for autoreactive B cells and potentially overcoming check points in B cell development and allowing for the breakage of tolerance [50]. In an agreeing study, apoptotic cells in germinal centers and apoptotic cells after UV exposure of skin in some SLE patients accumulated and auto-reactive B cells gained said reactivity in germinal centers through accumulation of apoptotic material on the follicular dendritic cell surface from impaired clearance and release of danger signals in the presence of modified self-antigens encouraged autoimmunity [51].

High mobility group box protein 1 (HMGB1) is a protein involved in chromosomal structure and can act as a proinflammatory mediator that stays in nucleosomes throughout apoptosis *in vitro*, and complexes of HMGB1 and nucleosomes have been detected in SLE patients [52]. Nucleosomes with HMGB1 from apoptotic cells were able to trigger the release of interleukin 1β, interleukin 6, interleukin 10, tumor necrosis factor alpha, induce expression of costimulatory proteins in macrophages and dendritic cells, and induce an autoantibody response to doublestranded DNA and histones in a Toll-like receptor 2 fashion [52]. In active SLE, especially those with renal involvement, HMGB1 levels were higher than healthy controls and were associated with a high SLE disease activity index, proteinuria, anti-double-stranded DNA autoantibodies, decreased levels of the complement protein C3, and the presence of high levels of anti- HMGB1 autoantibodies [53]. HMGB1 serum levels do not decrease after immunosuppressive therapy and may be indicative of continued inflammation [54].

Alpha-actinin is an acidic actin binding protein that helps maintain glomerular filtration and its expression is induced in lupus nephritis. Anti-alpha-actinin antibodies bound where anti-DNA antibodies had deposited in mouse models, indicating a potential role of protein-nucleic acid antigenic mimicry in renal damage [55]. Increased levels of anti-alpha-actinin antibodies were observed in sera in active nephritis, and there were some anti-DNA antibodies capable of cross reacting with alpha-actinin in mouse models [56]. Mice deficient in alpha-actinin-4 developed proteinuria, glomerular disease, and died after months, and it appears that alpha-actnin-4 plays a role in normal glomerular function and regulation of cellular motility as measured by increased lymphocyte chemotaxis in the absence of functional alpha-actinin- 4 [57]. In human SLE patients, the presence of anti-alpha-actinin antibodies are usually seen at high levels before or in the beginning of lupus nephritis, and the presence of anti-alpha-actinin antibodies are correlated with anti-dsDNA reactivity [58]. Anti-double-stranded DNA antibodies usually bindalpha-actinin, can bind mesangial cells and glomeruli *ex vivo*, and glomerular binding is not inhibited by DNase treatment but can be interrupted by alpha-actinin, indicating a role in cross-reactivity and potential induction of lesions in lupus nephritis [59]. In mouse models, two isoforms of alpha-actinin, alpha-actinin 1and alpha-actinin 4 can be targeted by anti-alpha-actinin antibodies, and enhanced alpha-actininexpression was observed in mesangial cells of lupus prone strains of mice, potentially allowing for increased antibody deposition [60]. In humans, anti-alpha-actinin antibodies correlate with glomerulonephritis, but whether they have predictive value for the development of SLE complications is not confirmed [61]. Nonautoimmune mice injected with alpha-actinin developed high levels of anti-nuclear autoantibodies, anti-chromatin autoantibodies, hypergammaglobulinemia, renal immunoglobulin deposition, and proteinuria, and the nephritogenic antibodies had higher affinities for alpha-actinin, chromatin, HMGB, and heat shock protein 70 potentially indicating commonalties between the antigens recognized by the antibodies [62].

## Roles of the Adaptive Immune System in Lupus Nephritis

Toll-like receptors are pattern-recognition receptors of the innate immune system that can subsequently aid in the activation of the adaptive immune system [63,64]. Toll-like receptors 3, 7, 8, and 9 are found within the endoplasmic reticulum, endosomes, or lysosomes and can recognize nucleic acids [65]. B cell receptors are capable of recognizing the Fc-regions of autoantibodies found in immune complexes, the immune complexes are endocytosed, and toll like receptors such as 7 and 9 can recognize the contents of the endosome, which may aid in the breakage of peripheral B cell tolerance [65]. Ligands of toll like receptor 7 like ribonucleoproteins can also stimulate plasmocytoid dendritic cells [65]. Toll like receptors can stimulate dendritic cell maturation and properties of said dendritic cells can tip the balance between T cell tolerance or activation [66]. Toll like receptors can be found in T cells and T regulatory cells, and stimulation of T cells by toll like receptors can enhance T cell immunity [66].

Toll-like receptor 9 recognizes DNA and is necessary for the spontaneous production of DNA autoantibodies by B-cells in mouse models but cannot solely induce lupus nephritis [63]. In the absence of toll-like receptor 7 in mouse models, which recognizes single-stranded RNA, antibodies against antigens with RNA, such as the Smith (Sm) antigen, could not be generated [63]. In mouse models lacking toll-like receptor 9 lymphocytes and plasmacytoid dendritic cells were hyper-activated and certain antibody (RNA-reactive) and interferon alpha levels (through plasmacytoid dendritic cell activation) were markedly increased in the serum, whereas absence of toll-like receptor 7 led to reduced activation of lymphocytes and decreased antibody levels in the serum, indicating opposing actions in inflammation [63,65].

**Figure 2:**
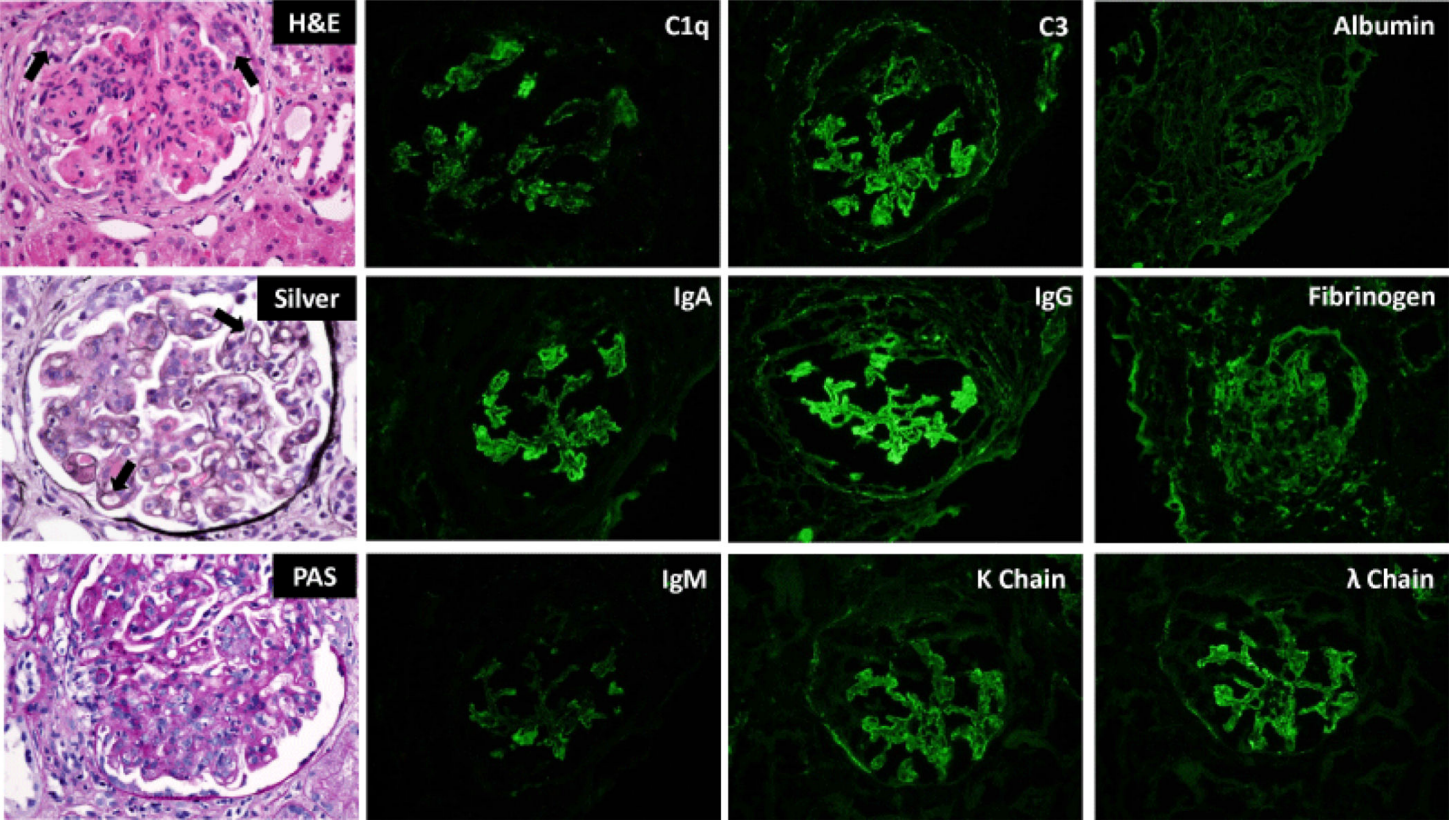
Characteristic histopathologic manifestations of diffuse proliferative lupus nephritis (Class IV-G/A by the International Society of Nephrology/Renal Pathology Society Classification). Glomeruli (left column) are diffusely hyper-cellular. Peripheral capillary loop lumens are compromised due to endocapillary proliferation (arrow). Large immune complex deposits are observed between layers of basement membrane material (silver stain, middle, arrow) giving rise to the characteristic “tram track” or double contour appearance of the capillary loops. Immunofluorescence studies demonstrate the characteristic “full house” pattern of positive staining for all immune reactants (IgG, IgA, IgM, C1q, C3, kappa and lambda light chain) in glomeruli.

In lupus nephritis, interferon gamma and interleukin 17 are elevated, potentially indicating that Th1 and Th17 cells may play a role in the severity of lupus nephritis [67]. FOXP3 levels are elevated as well, though this is believed to be compensatory [67]. Also, T regulatory cells may be converted to Th17 cells under the inflammatory conditions of lupus nephritis [67]. Interleukin 33 belongs to the interleukin 1 family and with its receptor ST2, it is able to induce a Th2 response [68]. By inhibiting interleukin 33 with an antibody in MRL/lpr mice, proteinuria was decreased; anti-double-stranded DNA antibody levels were decreased in the serum; nephritis was reduced; Th17 cell numbers and interleukin 1β, interleukin 6, and interleukin 17 levels were decreased; circulating levels and deposition of immune complexes were decreased; T regulatory cells were increased; and myeloid-derived suppressor cells were increased, indicating that interleukin 33 inhibition may slow the progression of SLE by increasing the T regulatory cell and myeloid-deprived suppressor cell populations and inhibiting Th17 cells and pro-inflammatory responses [68]. In children with active lupus nephritis, intravenous methyl-prednisolone pulse therapy was shown to restore CD4^+^FoxP3^+^ and CD8^+^Foxp3^+^ T regulatory cell levels, and treated CD8^+^FoxP3^+^ T regulatory cells suppressed CD4^+^ T cell proliferation and induced their apoptosis, modulating autoimmune response in lupus nephritis [69]. The Ccr1 chemokine receptor is associated with leukocyte infiltration with NZB/W mice with nephritis being more responsive to Ccr1 ligand and subsequent T cell, mononuclear phagocytes, and neutrophil influx than pre-nephritic mice [70]. Treatment with a Ccr1 antagonist decreased accumulation of effector CD4^+^ T cells, memory CD4^+^ T cells, Ly6C^+^ monocytes, M1 macrophages, and M2 macrophages; decreased tubulointerstial and glomerular damage; slowed the onset of proteinuria; extended life-span; but had no therapeutic effect in slowing the infiltration of B cells, insinuating that Ccr1 is important for the recruitment of T cells and mononuclear phagocyte cells [70]. CD147 is a glycosylated transmembrane protein that plays a role in regulating lymphocyte response and leukocyte influx, it is induced in injured glomeruli and incoming inflammatory cells, and plasma levels reflect histological disease activity, making it a good potential biomarker in lupus nephritis patients [71] (Figure 2). The presence of greater than or equal to 800/100 mL of CD4^+^ T cells in urine is a sensitive and specific biomarker for active proliferative nephritis in SLE patients [72].

## Conclusions

This review examined the roles of complement, autoantibodies and apoptosis, and the adaptive immune system in contributing to the pathogenesis of lupus nephritis. Complement aids in opsonizing immune complexes for degradation by effector immune cells, and deficiency in early components of the complement cascade are associated with active disease [3,5]. Inefficient apoptotic clearing can allow molecules normally contained within cells to become accessible to autoantibodies and the breaking of tolerance can occur. The innate immune system may induce the adaptive immune system through toll like receptors by activating auto-reactive B cells and instigating a T cell response. Products of the adaptive immune response may serve as markers for disease activity and blocking activation of specific components of the adaptive immune may have therapeutic effects in controlling inflammation. Lupus nephritis is a serious manifestation of SLE with a complex pathogenesis, but hopefully improved understanding of its pathogenesis can result in the continued development of more specific and effective treatments.
